# Characterization of Chemically-Induced Bacterial Ghosts (BGs) Using Sodium Hydroxide-Induced *Vibrio parahaemolyticus* Ghosts (VPGs)

**DOI:** 10.3390/ijms17111904

**Published:** 2016-11-15

**Authors:** Hyun Jung Park, Sung Oh, Nagarajan Vinod, Seongmi Ji, Han Byul Noh, Jung Mo Koo, Su Hyeong Lee, Sei Chang Kim, Ki-Sung Lee, Chang Won Choi

**Affiliations:** 1 Department of Biology & Medicinal Science, Pai Chai University, Daejeon 35345, Korea; parkhj0524@pcu.ac.kr (H.J.P.); 5star@pcu.ac.kr (S.O.); biovinz@gmail.com (N.V.); dongny12@pcu.ac.kr (S.J.); creator1018@pcu.ac.kr (H.B.N.); jungmo9@gmail.com (J.M.K.); kimsc@pcu.ac.kr (S.C.K.); kslee@pcu.ac.kr (K.-S.L.); 2 Cheongshim International Academy, Gapyeong-gun, Gyeonggi-do 12461, Korea; potatoclsrn@naver.com

**Keywords:** bacterial ghosts (BGs), *Vibrio parahaemolyticus*, chemically-induced lysis, minimum inhibition concentration (MIC), sodium hydroxide (NaOH), lipopolysaccharides (LPS), endotoxic activity, macrophages, cytotoxicity, cytokine

## Abstract

Acellular bacterial ghosts (BGs) are empty non-living bacterial cell envelopes, commonly generated by controlled expression of the cloned lysis gene *E* of bacteriophage PhiX174. In this study, *Vibrio parahaemolyticus* ghosts (VPGs) were generated by chemically-induced lysis and the method is based on minimum inhibitory concentration (MIC) of sodium hydroxide (NaOH), acetic acid, boric acid, citric acid, maleic acid, hydrochloric acid, and sulfuric acid. The MIC values of the respective chemicals were 3.125, 6.25, <50.0, 25.0, 6.25, 1.56, and 0.781 mg/mL. Except for boric acid, the lysis efficiency reached more than 99.99% at 5 min after treatment of all chemicals. Among those chemicals, NaOH-induced VPGs appeared completely DNA-free, which was confirmed by quantitative real-time PCR. Besides, lipopolysaccharides (LPS) extracted from the NaOH-induced VPGs showed no distinctive band on SDS-PAGE gel after silver staining. On the other hand, LPS extracted from wild-type bacterial cells, as well as the organic acids-induced VPGs showed triple major bands and LPS extracted from the inorganic acids-induced VPGs showed double bands. It suggests that some surface structures in LPS of the NaOH-induced VPGs may be lost, weakened, or modified by the MIC of NaOH. Nevertheless, *Limulus amoebocyte* lysate assay revealed that there is no significant difference in endotoxic activity between the NaOH-induced VPGs and wild-type bacterial cells. Macrophages exposed to the NaOH-induced VPGs at 0.5 × 10^6^ CFU/mL showed cell viability of 97.9%, however, the MIC of NaOH did not reduce the cytotoxic effect of wild-type bacterial cells. Like *Escherichia coli* LPS, the NaOH-induced VPGs are an excellent activator of pro-inflammatory cytokines (IL-1β and iNOS), anti-inflammatory cytokine (IL-10), and dual activities (IL-6) in the stimulated macrophage cells. On the other hand, the induction of TNF-α mRNA was remarkable in the macrophages exposed with wild-type cells. Scanning electron microscopy showed the formation of trans-membrane lysis tunnel structures in the NaOH-induced VPGs. SDS-PAGE and agarose gel electrophoresis also confirmed that cytoplasmic proteins and genomic DNA released from the VPGs to culture medium through the lysis tunnel structures. Taken together, all these data indicate that the NaOH-induced VPGs show the potency of a safe, economical, and effective inactivated bacterial vaccine candidate.

## 1. Introduction

Vaccines are very efficient in protecting human and animal hosts from bacterial pathogens. Nevertheless, their roles against infections are limited because currently available vaccines are usually serotype- or species-specific [1]. Therefore, it is necessary for the development of cross-protective vaccines against infectious diseases. Among various potential candidates, bacterial ghosts (BGs) have been proposed as a multivalent vaccine candidate [2]. A typical method to produce BGs is by controlled expression of the cloned lysis gene *E* of bacteriophage PhiX174 [3], which forms trans-membrane lysis tunnel structures on the bacterial cell surfaces [4,5,6,7]. The resultant BGs are non-living whole cell envelopes lacking cytoplasmic contents but retaining basic bacterial cell surface structures such as lipopolysaccharides (LPS), lipids, and peptidoglycan [8,9,10]. These functional and antigenic determinants possessed the intrinsic adjuvant properties and induced both humoral and cell-mediated immune responses against virulent challenge in various animal models [8,11]. Furthermore, *Vibrio cholrae* BGs induced antibodies showing vibriocidal activity and these antibodies provided protection from homologous and heterologous challenges in experimental animals [4,5].

Although the lysis *E* gene-induced BGs provided efficient protection against specific infections [12,13,14], a primary disadvantage of the method is a limitation to Gram-negative bacteria only. Secondly, it is very difficult to reach 100% lysis rate of BGs strain in a short time [4], which may cause potential risks. Thirdly, it is a multi-step process that is cost expensive and time consuming. Alternatively, Amara et al. [15] demonstrated the protocol for *Escherichia coli* BGs preparation using the minimum inhibition concentration (MIC) and the minimum growth concentration (MCG) of various chemicals. Recently, sodium hydroxide (NaOH)-induced BGs were generated from a Gram-negative bacterium (*Salmonella enteriditis*) [16] and a Gram-positive bacterium (*Staphylococcus aureus*) [17]. Nevertheless, it has never been characterized as to why NaOH was chosen as the best chemical to produce BGs. In addition, the question arises as to whether the chemically-induced BGs maintain LPS on their cell envelope in the same way as untreated wild-type bacterial cells, because alkaline hydrolysis is known as a depyrogenation method that destroys the 8 carbon sugar: 2-keto-3-deoxyoctonic acid that links Lipid-A to the core polysaccharide [18].

To address the questions, we selected a Gram-negative and halophilic bacterium, *V. parahaemolyticus* that has been recognized as an opportunistic pathogen to both humans and marine animals. It causes acute gastroenteritis after direct consumption of contaminated raw seafood [19], or life-threatening septicemia to patients with a preexisting medical condition [20]. It can produce a capsule with a number of different somatic (O) and capsular (K) antigens [21]. More than 80 serotypes of *V. parahaemolyticus* have been described worldwide, based on the O and K antigenic properties [22]. To date, some BGs have been produced from other *Vibrio* species (*V. cholera*, *V. anguillarum*, *V. vulnificus*) by using the lysis *E*-gene system [4,5,23,24], but no BGs have been reported from *V. parahaemolyticus*. In the present study, we determined the MIC of various chemicals (six acids and one alkali) on *V. parahaemolyticus* cells and characterized their efficiencies to produce *V. parahaemolyticus* ghosts (VPGs). Especially, we focused on the generation time and the presence or absence of DNA in the chemically-induced VPGs. For the determination of the endotoxic potential, we investigated the effect of NaOH on LPS extracted from the VPGs qualitatively by SDS-PAGE/silver staining and quantitatively by *Limulus* amoebocyte lysate (LAL) assay. Furthermore, we also investigated the cytotoxic effect of the NaOH-induced VPGs on murine macrophages RAW 264.7 cells and their immunomodulatory activities using mRNA expression of pro-inflammatory and anti-inflammatory cytokines. In the end, we confirmed that cytoplasmic proteins and denatured DNA released from the NaOH-induced VPGs to culture medium through the trans-membrane lysis tunnel structure formed on the surface of a cell envelope. Our results indicate that the NaOH-induced VPGs show potential for a safe, cheap, and effective inactivated bacterial vaccine.

## 2. Results and Discussion

### 2.1. Effects of Chemicals on Bacterial Cell Envelopes

Numerous chemicals are known to inhibit bacterial cell growth due to their adverse effects on the integrity of bacterial cell envelopes. Especially, acids and alkalis have strong bactericidal effects. The MICs of six acids (acetic acid, boric acid, citric acid, hydrochloric acid, maleic acid, sulfuric acid) and 1 alkali (NaOH) against *V. parahaemolyticus* were determined by the two-fold broth dilution method (Table 1 and Figure S1). Furthermore, no colony was formed on LB agar plates spread with *V. parahaemolyticus* bacteria treated with respective chemicals at their MICs (Figure S2). This was consistent in all of the three replicates performed. Among those chemicals, hydrochloric acid showed the lowest MIC, while boric acid showed the highest MIC. Due to high MIC value, boric acid was excluded from a further study. Previously, acetic acid, hydrochloric acid, and lactic acid inhibited *Helicobacter pylori* growth in a pH-dependent manner [25], while butyric acid inhibited the bacterial growth in a pH-independent manner [26]. In this study, the MICs of different chemicals changed the culture medium pH from pH 7.0 to 9.99 (NaOH), 4.28 (acetic acid), 3.11 (citric acid), 3.95 (hydrochloric acid), 3.56 (maleic acid), and 5.83 (sulfuric acid). The data indicated that the different chemicals above did not inhibit the *V. parahaemolyticus* growth in a pH-dependent manner but they inhibited the bacterial growth in a concentration-dependent manner. Figure S3 showed a complete lysis of the *V. parahaemolyticus* cells treated with different chemicals, respectively, at various time points. Except for acetic acid (99.99% at 60 min), other chemicals reached 100% lysis rate within 10 min. In the lysis *E* gene-mediated method, it took almost 8 h to produce non-living BGs with complete lysis [8] and the lysis efficiency was commonly 99.9% [3,27,28]. Thus, production of BGs by chemically-mediated lysis is a more simple and rapid process than that by the genetically engineered *E* gene-mediated lysis method. This finding revealed the first difference between the chemically-induced BGs and the lysis *E* gene-induced BGs.

### 2.2. Determination of DNA-Free Vibrio parahaemolyticus Ghosts (VPGs) by Agarose Gel Electrophoresis and qPCR

**Figure 1:**
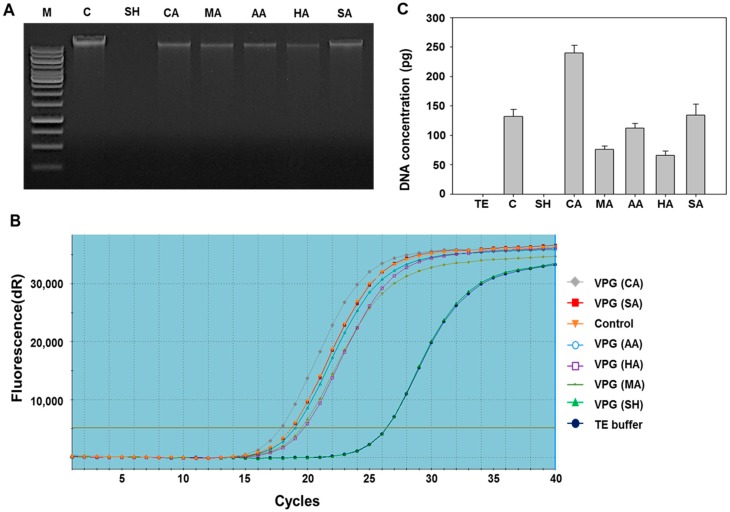
(**A**) Agarose gel (1%) electrophoresis of genomic DNA extracted from *Vibrio parahaemolyticus* ghosts (VPGs) treated with MICs (minimum inhibitory concentration) of sodium hydroxide (SH), citric acid (CA), maleic acid (MA), acetic acid (AA), hydrochloric acid (HA), and sulfuric acid (SA), respectively, for 60 min. M: 1 kb DNA ladder and C: untreated *V*. *parahaemolyticus*; (**B**) Quantitative analysis of DNA content extracted from VPGs treated with MICs of respective chemicals for 60 min using real-time PCR with SyBr Green detection system; (**C**) DNA quantity of respective VPGs was compared with untreated *V*. *parahaemolyticus* (positive control) and TE-buffer (negative control). A standard curve for absolute quantification of bacterial DNA was obtained by diluting a genomic DNA of *V. parahaemolyticus* in a solution containing 1 ng DNA.

The worldwide occurrence of *V. parahaemolyticus* in human hosts is associated with its virulence factors, such as hemolysin (TDH) [29] and TDH-related hemolysin (TRH) [30]. Almost clinical strains of *V. parahaemolyticus* have β-hemolytic activity ascribed to *tdh* and *trh* genes and the activity causes the Kanagawa phenomenon [31]. In this regard, a careful consideration of chemicals needed to induce complete DNA-free BGs is necessary. In this study, only NaOH-induced VPGs showed a complete absence of genomic DNA on agarose gel, while VPGs induced by other chemicals showed a distinctive presence of genomic DNA (Figure 1A). To further confirm this, we conducted qPCR analysis with VPGs induced by the respective chemicals. Amplification of partial ribosome DNA was found in all acids-induced VPGs, while complete DNA-free VPGs were observed in the NaOH-induced VPGs (Figure 1B,C). It suggests that acidic pH of the culture medium under given chemicals and their concentrations could not be effective to remove DNA completely from the VPGs. In general, DNA can be cleaved into nucleosides and nucleotides under acidic pH <3 that disrupts phosphodiester bonding. Moderate or high concentrations of alkali cause deprotonation, hydrogen bonding disruption between base pairs, and hydrolysis of the phosphodiester bonds of DNA. Considering the alkali effect on DNA, our data indicate that NaOH causes genomic DNA to be cleaved into small fragments which can be expelled completely from the VPGs. Previous studies also showed that the genomic DNA of a Gram-negative bacterium [16] and a Gram-positive bacterium [17] was completely devoid from respective BGs treated with NaOH.

### 2.3. Analysis of Lipopolysaccharides (LPS) Profile in Chemically-Induced VPGs

LPS molecule contains three distinct regions: hydrophobic lipid-A region, core oligosaccharide region, and O-Ag polysaccharide region. The lipid-A is a key constituent showing endotoxic activities, while the O-Ag polysaccharide provides the major antigenic variability of a cell surface and produces vibriocidal antibodies in a host [32]. Previously, the LPS profile of *V*. *chlolerae* showed mitogenic effects, adjuvant and antigenic properties, hemagglutinating activity, and several endotoxic activities such as pyrogenicity, lethality to mice, local Shwartzmann reaction, and limulus lysate gelation [32]. Therefore, LPS can be a crucial problem of vaccines prepared from Gram-negative bacteria and the LPS removal is a great concern. However, a previous study showed that tolerable doses of BGs can induce efficient immune responses without leading to LPS-related side effects, such as fever, in experimental animals [33].

**Figure 2:**
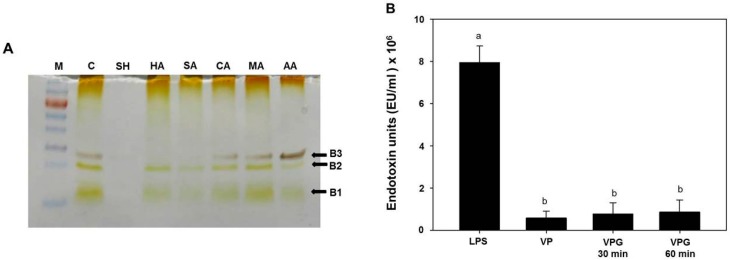
(**A**) Silver stained SDS-PAGE gel showing banding patterns of lipopolysaccharides (LPS) extracted from different chemical-treated VPGs. Lanes M (molecular weight marker), C (untreated *V*. *parahaemolyticus* cells), SH (sodium hydroxide), HA (hydrochloric acid), SA (sulfuric acid), CA (citric acid), MA (maleic acid), and AA (acetic acid). The amounts of LPS were loaded were 5 μL for each lane; (**B**) Endotoxic activity of the LPS (5 μg/mL) from *Escherichia coli*, *V. parahaemolyticus* wild-type cells (VP), VPGs treated with NaOH for 30 and 60 min. The endotoxic activity was compared using the LAL assay. The LPS of *E*. *coli* 011:B4 was used as a standard and results are given in endotoxic units (EU). All data are expressed as the mean ± the standard error of the mean. Mean separation by Duncan’s multiple range test at *p* < 0.05. The same letter above bars represents no significant difference between treatments.

In order to determine the effect of NaOH MIC on LPS, we extracted LPS from the NaOH-induced VPGs and wild-type *V. parahaemolyticus* cells, respectively, and compared its banding profile using SDS-PAGE with silver staining. In a previous study, LPS profiles of *V*. *parahaemolyticus* consisted of triplet bands (named B1, B2, and B3) on SDS-PAGE gel, and slower-migrating bands (B2 and B3) were proven as aggregates of a fast-migrating band (B1) by re-electrophoresis. This banding pattern indicated that *V*. *parahaemolyticus* LPS does not have an 0-specific side chain [34]. Additionally, LPS isolated from the lysis *E* gene-induced *V. cholrae* BGs and wild-type bacterial preparations showed the same banding profile on silver-stained SDS-PAGE gel [4]. In this study, we also observed three major bands in LPS extracted from the wild-type *V*. *parahaemolyticus*, as well as organic acids (citric acid, maleic acid, and acetic acid)-induced VPGs (Figure 2A, lanes CA, MA, and AA). Double bands (B1 and B2) were observed in LPS extracted from inorganic acids (hydrochloric acid and sulfuric acid)-induced VPGs (Figure 2A, lanes HA and SA), while no distinctive band was observed in LPS extracted from the NaOH-induced VPGs (lane SH). It suggests that some surface structures in the LPS of the VPGs may be lost, weakened, or modified by treatment with the MIC of NaOH. This finding revealed the second difference between the chemically-induced BGs and the lysis *E* gene-induced BGs.

Free LPS is known to be more toxic than membrane-bound LPS [35]. In experimental animals, much higher LPS concentrations were tolerated when LPS was associated to BGs than when it was in a free form [33]. To determine the free endotoxic activities quantitatively, we performed LAL assay with LPS extracted from the NaOH-induced VPGs and wild-type bacterial cells, respectively. LPS (5 μg/mL) from *Escherichia coli* (a positive control) was measured at 8 × 10^6^ endotoxic units (EU), while the LPS preparations from the VPGs treated with NaOH for 30 and 60 min showed 9.6%–10.9% of the endotoxic activity of the *E*. *coli* LPS. The endotoxic activities of LPS extracted from the VPGs were slightly higher than that of LPS extracted from their corresponding wild-type cells, but there was no significant difference (Figure 2B). We speculate that the MIC of NaOH was not enough for the depyrogenation of LPS from the VPGs. Otherwise, the level of endotoxin may initially increase as part of the separation process by alkaline hydrolysis, as described previously [18].

### 2.4. Comparison of Cytotoxicity Tests for VPGs

**Figure 3:**
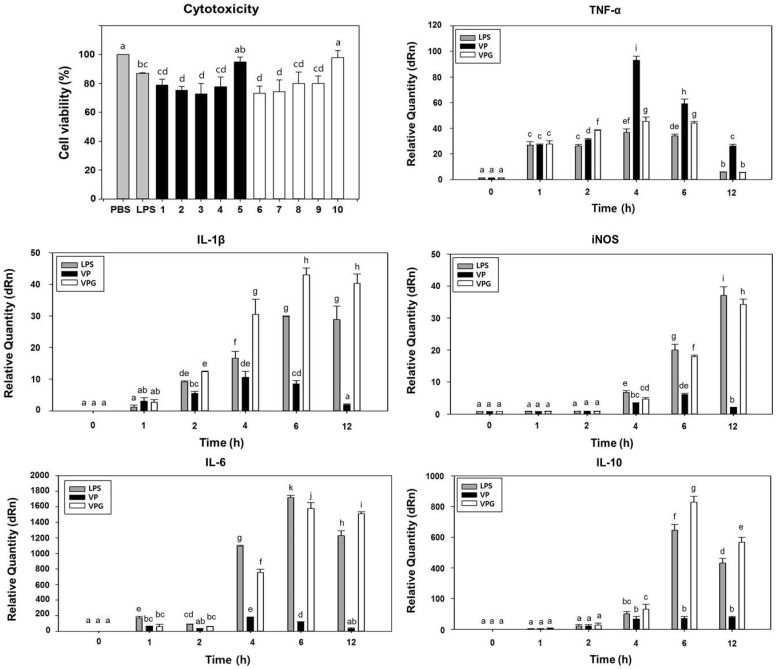
VPGs-exposed murine macrophage (RAW 264.7) shows cell viability and stimulates both pro- and anti-inflammatory cytokine production. To determine cytotoxicity, macrophages were exposed to PBS buffer, LPS from *Escherichia coli*, *V. parahaemolyticus* wild-type cells (bars 1–5), and VPGs treated with NaOH for 60 min (bars 6–10), respectively. At 24 h post-exposure, macrophages were collected for analysis of cell viability using Cell counting Kit-8. Bars represent exposure doses of 2.5 × 10^6^ (1 and 6), 1.7 × 10^6^ (2 and 7), 1.3 × 10^6^ (3 and 8), 1.0 × 10^6^ (4 and 9), and 0.5 × 10^6^ (5 and 10) CFU/mL, respectively. Absorbance was measured at 450 nm and all experiments were performed in triplicate. Cytotoxic activity is expressed as the percentage of cell viability by the formula described in Materials and Methods. At 4 h post-exposure with LPS from *Escherichia coli*, *V. parahaemolyticus* wild-type cells (VP), and NaOH-induced VPGs for 60 min, respectively, macrophages were collected for analysis of gene expression for cytokines TNF-α; IL-1β; iNOS; IL-6; and IL-10 using RT-qPCR. Data are representative of triplicate experiments with each sample run in triplicate. All data are expressed as the mean ± the standard error of the mean. Mean separation by Duncan’s multiple range test at *p* < 0.05. The same letter above bars represented no significant difference between treatments.

In a previous study, *V*. *parahaemolyticus* LPS was 200-fold less toxic than *Salmonella typhimurium* LPS and the LPS was detoxified significantly by γ-radiation, which caused a reduction in the cytotoxicity [36]. In this study, the cytotoxicity was compared using the viability of RAW 264.7 murine macrophages exposed with the NaOH-induced VPGs and wild-type bacterial cells, respectively (Figure 3). LPS of *E*. *coli* included in this analysis showed cell viability of 87.1% under the given concentration. The macrophages exposed with decreasing concentration of the VPGs showed increasing cell viability, which was similar to those exposed with wild-type bacterial cells under the same range of concentration. It suggests that the MIC of NaOH did not completely reduce the cytotoxic effect of wild-type bacterial cells. The macrophages exposed to the VPGs at 0.5 × 10^6^ CFU/mL showed the maximum cell viability (97.9%), while those exposed to the VPGs at 2.5 × 10^6^ CFU/mL showed the minimum cell viability (73.1%). Because the VPGs and wild-type cells provided the maximum cell viability at a concentration of 0.5 × 10^6^ CFU/mL, we used this concentration in the following cytokine experiments.

In a previous study, alkaline treatment to a Gram-negative bacterial LPS resulted in reduced toxicity and the deacylated LPS and showed that ester-linked fatty acids were eliminated, while amide-linked fatty acids were kept intact [37]. The resultant LPS was antigenically poor [37,38] or deficient [39]. However, our previous studies demonstrated that immunization with the NaOH-induced BGs from a Gram-negative bacterium and a Gram-positive bacterium induces effective immune responses and provides a good protection against virulent challenge [16,17]. It suggests that the NaOH treatment does not affect the immunogenicity of VPGs or their potential as a vaccine candidate. Presumably, it is possible to assign that the immunogenicity of the NaOH-induced BGs could be derived from other cell envelope components rather than LPS. In the lysis *E* gene-induced BGs, LPS associated to the BG envelope was not altered during the production process [33]. This finding revealed the third difference between the chemically-induced BGs and the lysis *E* gene-induced BGs.

### 2.5. Induction of Cytokine Gene Expression in Murine Macrophages-Exposed VPGs

LPS does not act directly against cells or organs but through activation of the immune cells, especially through monocytes and macrophages, with the production of various cytokines. It has been reported that macrophages infected with *V*. *parahaemolyticus* produce a classical innate immune activation response characterized by pro- and anti-inflammatory cytokine gene expression [40]. Therefore, we determined the activation of pro-inflammatory cytokines, such as tumor necrosis factor (TNF)-α, interleukin (IL)-1β and inducible nitric oxide synthase (iNOS), anti-inflammatory cytokine (IL-10) and both of properties (IL-6) in the macrophages exposed to the NaOH-induced VPGs, wild-type bacterial cells, and *E*. *coli* LPS, respectively. In this study, TNF-α mRNA induction was highly induced in the macrophages exposed to the wild-type bacterial cells in a time-dependent manner and its maximum level was found at 4 h. This maximum level was 2.5- and 2.0-fold higher than that in the macrophages exposed to the *E*. *coli* LPS and VPGs, respectively (Figure 3). IL-1β is known as a key mediator of the inflammatory response and a pro-inflammatory cytokine that is pivotal for host–defense responses to infection and injury [41]. In the macrophages exposed to the VPGs, the induction of IL-1β mRNA was highly induced in a time-dependent manner and its maximum level was found at 6 h. This maximum level was 1.4 and 5.0-fold higher than that in the macrophages exposed to the *E*. *coli* LPS and wild-type bacterial cells, respectively (Figure 3). Nitric oxide (NO) plays a key role for the host innate immune response to pathogens [42] and in the regulation of certain physiological functions [43]. Despite beneficial roles of NO, its excessive production leads to some inflammatory diseases [44]. In murine macrophage RAW 264.7 cells, the LPS stimulation alone can induce iNOS transcription, which controls NO production [45]. In this study, the macrophages exposed to the *E*. *coli* LPS also showed the highest iNOS mRNA expression at 12 h. Like the *E*. *coli* LPS, the maximum level of iNOS mRNA in the macrophages exposed to the VPGs was found at 12 h, which was 17.3-fold higher than that in the macrophage exposed to the wild-type bacterial cells (Figure 3).

IL-6 has pro- and anti-inflammatory properties and is known to be a multifunctional cytokine that regulates immune responses, bone homeostasis, metabolism, and inflammation [46,47]. In this study, the mRNA expression of IL-6 reached the highest level at 6 h in the macrophages exposed to the *E*. *coli* LPS. Similarly, the maximum level of IL-6 mRNA in the macrophages exposed to the VPGs was found at 6 h, which was 13.9-fold higher than that in the macrophages exposed to the wild-type bacterial cells (Figure 3). IL-10 is another pleiotropic cytokine that modulates the function of numerous adaptive immune-related cells. Although IL-10 was regarded as an immunosuppressive and anti-inflammatory cytokine, it has immunostimulatory properties, including the ability to activate T cells, B cells, NK cells, and mast cells [48]. In a previous study, IL-10 mRNA was upregulated and significantly increased in RAW.264.7 macrophages infected with *V*. *parahaemolyticus* [40]. In this study, the mRNA expression of IL-10 was slightly induced in the macrophages exposed to the wild-type bacterial cells, whereas the mRNA expression of IL-10 was highly induced in the macrophages exposed to the VPGs. The maximum level of IL-10 mRNA was reached at 6 h in the macrophages exposed to the VPGs, which was 1.3- and 11.7-fold higher than that in the macrophages exposed to the *E*. *coli* LPS and wild-type cells, respectively (Figure 3). Taken together, our data indicated that the VPGs can activate macrophages to secrete both pro-inflammatory and anti-inflammatory cytokines.

### 2.6. Morphological Observation of NaOH-Induced VPGs by Scanning Electron Microscopy (SEM)

**Figure 4:**
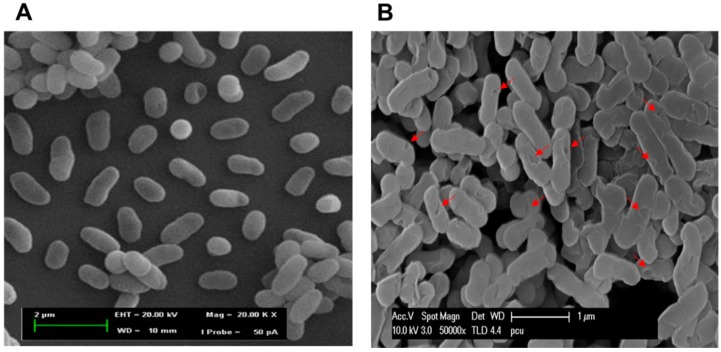
Scanning electron microscopic analysis of VPGs. (**A**) Untreated control shows intact *V. parahaemolyticus* bacterial cells; and (**B**) MIC of NaOH-induced VPGs. The small arrows show the trans-membrane lysis tunnels.

SEM showed that the NaOH-induced VPGs maintained the basic cell morphology of bacteria, but displayed surface modification of the cell envelope. When compared with an electron micrograph of untreated wild-type cells (Figure 4A), an electron micrograph of VPGs showed the formation of trans-membrane lysis tunnel structure on the surface of VPGs (Figure 4B, arrowheads). It indicated that the morphology of VPGs is not affected by the lysis process except for the formation of tunnel structures. Most importantly, three-day-old cultures of *V*. *parahaemolyticus* maintained resilient strength of cell walls, which would be sufficient to create holes to evacuate cytoplasmic and genetic contents in bacterial cells. This is in agreement with previously reported chemically-induced BGs from *H*. *pylori* [26], *E*. *coli* [15], *Salmonella enteriditis* [16], and *Staphylococcus aureus* [17].

### 2.7. Analysis of Protein and DNA Profiles in Sodium Hydroxide-Induced VPGs

**Figure 5:**
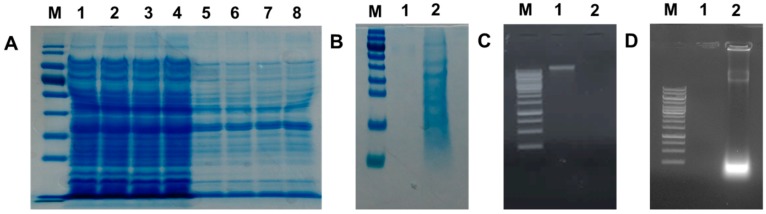
Characterization of culture pellets and supernatants of *V. parahaemolyticus* PCU-1 treated with MIC of NaOH. (**A**) Total proteins extracted from culture pellets of untreated bacterial cells (lanes 1–4: 15, 30, 45, and 60 min) and NaOH-treated VPGs (lanes 5–8: 15, 30, 45, and 60 min). M: Protein Marker; (**B**) Total proteins extracted from culture supernatants of untreated (lane 1) and NaOH-treated bacterial cells for 60 min (lane 2). Agarose gel (1%) electrophoresis of culture pellets containing VPGs (**C**); and culture supernatants (**D**). M: 1 kb Marker Ladder, lane 1: untreated *V. parahaemolyticus* PCU-1 (60 min) and lane 2: NaOH-treated *V. parahaemolyticus* PCU-1 (60 min).

Remaining protein content of the NaOH-induced VPGs (15, 30, 45, and 60 min) was evaluated by SDS-PAGE analysis. In comparison with untreated control cells (Figure 5A, lanes 1–4), the NaOH-treated bacterial cells (lanes 5–8) showed weaker protein band intensities. This is due to lack of cytoplasmic contents in the NaOH-induced VPGs. Moreover, a spectrophotometer was used to estimate the protein concentration of VPGs to compare with that of the untreated control cells. The amount of protein concentration in the untreated cells was much higher than that of the VPGs (data not shown). As expected, cytoplasmic proteins released from the VPGs were found in the culture supernatants (Figure 5B). It suggests that cytoplasmic proteins are released from the VPGs through the trans-membrane tunnel structure observed by SEM.

Agarose gel electrophoresis was also used to confirm the release of genome DNA from the NaOH-induced VPGs into culture medium through the trans-membrane tunnel structure. The absence of genomic DNA was found from the NaOH-induced VPGs (Figure 5C, lane 2) when compared to the wild-type cells that clearly showed the DNA band (lane 1). As expected, denatured genomic DNA by alkaline lysis was released into culture medium and found in the culture supernatants (Figure 5D, lane 2). The results support that the osmotic pressure difference between the cytoplasm and the surrounding medium could be the driving force for the rapid release of the cytoplasmic content through the trans-membrane tunnel structure [9]. Similarly, cytoplasmic proteins and DNA of butyric acid-treated *H*. *pylori* were recovered from the extracellular environment, supporting that the chemical agent creates holes which lead to evacuation of cytoplasmic and genetic contents in bacterial cells [26].

## 3. Materials and Methods

### 3.1. Bacterial Strain and Culture Condition

A Gram-negative bacterium *V. parahaemolyticus* PCU-1 (Department culture collection) was used to produce non-living BGs. The bacterial culture was freshly grown in Luria-Bertani (LB) broth (pH 7.0) at 37 °C in a shaking incubator at 200 rpm. The bacterial cell growth and lysis were monitored by measuring the absorbance spectrophotometrically at 600 nm (OD_600_). The CFU was determined as described previously [49].

### 3.2. Chemical Agents and Determination of Their MICs (Minimum Inhibitory Concentration)

Acetic acid, boric acid, citric acid, hydrochloric acid, maleic acid, sulfuric acid, and sodium hydroxide were purchased from Sigma-Aldrich (St. Louis, MO, USA). MICs of the different chemical agents were determined by using the two-fold broth dilution method, as described previously [16,17]. The *V. parahaemolyticus* culture was grown in LB medium and adjusted at a final concentration of 1 × 10^6^ CFU/mL. Serially diluted solution of the six chemicals (stock solution, 50 mg/mL), respectively, was added to the bacterial culture and then incubated at 37 °C for 18 h. After incubation, the MICs of different chemicals were determined in triplicate. To confirm the MICs, the culture that showed no visible growth was verified by spreading 100 µL of the culture onto LB agar plates and incubated at 37 °C for 24 h.

### 3.3. Production of VPGs

The 72 h cultured biomass of *V. parahaemolyticus* was centrifuged at 10,000× *g* for 10 min at 4 °C and the bacterial pellets were collected, washed twice with phosphate-buffered saline (PBS, 5 mM K_2_HPO_4_, 5 mM KH_2_PO_4_, 150 mM NaCl, pH 7.0), and adjusted at a final concentration of 1 × 10^6^ CFU/mL. One mL of stock solutions (5×) of the different chemicals prepared by MIC values, respectively, was added to 2 mL of the bacterial suspension. Subsequently, the sterilized distilled water (2 mL) was added to give a final concentration equal to 1× for each chemical. Thereafter, all the samples were incubated at 37 °C for 60 min. At different time points (15, 30, 45 and 60 min), the lysis rates of the untreated control and the bacterial samples treated with the respective chemicals were determined by standard plating procedure. The viability assay for each time was carried out in triplicate. After lysis had been completed, the chemically-induced VPGs were harvested by centrifugation (15 min, 4 °C, 10,000× *g*) and washed twice with PBS. The final cell pellets were resuspended in ice-cold PBS and stored at 4 °C until further use.

### 3.4. Analysis of DNA-Free VPGs by Real-Time PCR

To analyze the completely DNA-free VPGs in *V. parahaemolyticus* cells, the bacterial cells treated with the MICs of different chemicals, as well as untreated control cells, were collected at 15, 30, 45, and 60 min. Genomic DNA was prepared using a bacterial genomic DNA isolation kit (iNtRON Biotechnology, Seongnam-si, Gyeonggi-do, Korea), according to the manufacturer’s instructions. The extracted genomic DNA was analyzed by electrophoresis in 1% agarose gel and then qPCR assays were performed by using the SyBr Green detection system. The genomic DNA extracted from various lysis times was used as the template for qPCR. The 16S rRNA of *V. parahaemolyticus* was amplified with specific primers (Table 2). The total volume of each tube was 20 µL, containing 1 µL of 1:100 template DNA, 1 µL of both 16s rRNA forward and reverse primers (10 pM/µL), 10 µL of 2× SYBR Green QPCR Master Mix (Agilent Technologies, USA), and 7 µL of sterilized distilled water. Reaction was initiated at 95 °C for 10 min, followed by 40 cycles at 95 °C for 10 s, 55 °C for 40 s, and 72 °C for 30 s. The qPCR reaction was performed in a Stratagene Mx3000P QPCR machine (Agilent Technologies, Santa Clara, CA, USA). Negative control (TE buffer) and DNA extracted from the untreated bacterial cells were simultaneously included in each run. Each sample was quantified in triplicate and processed three different times with qPCR under the same conditions. Quantification of a relative amount of DNA in the respective chemicals-treated VPGs was calculated by comparing the *C*_t_ value of each sample to the *C*_t_ values of a standard curve. The standard curve for absolute quantification of bacterial DNA was obtained by diluting a genomic DNA of *V. parahaemolyticus* in a solution containing 1 ng DNA. The equation (*y* = −1.443 × log(*x*) + 25.808; *r^2^* = 0.9994) was obtained on the standard curve by plotting the *C*_t_ values over the logarithm of the amount of bacterial genomic DNA present in two-fold dilution series. The experiments were analyzed with auto-baseline and manual thresholds chosen from the exponential phase of the qPCR amplification. After the data analysis, the *C*_t_ number and DeltaRn (dRn) were used for statistical analyses. Data were analyzed using Mxpro software and the comparative threshold cycle (2^−ΔΔ*C*t^) method [50].

### 3.5. LPS Extraction, SDS-PAGE, and LAL Assay

LPS was extracted using an LPS extraction kit (iNtRON Biotechnology) according to the manufacturer’s protocol. Briefly, VPGs (5 mL culture) induced by various chemicals were centrifuged at 13,000 rpm for 5 min. The VPGs pellet was treated with 1 mL of supplied lysis buffer and vortexed vigorously. After adding 200 μL of chloroform, the mixture was centrifuged at 13,000 rpm for 10 min at 4 °C. Then, the supernatant (400 μL) was mixed well with a supplied purification buffer and incubated for 10 min at −20 °C. After centrifuging the mixture solution at 13,000 rpm for 15 min at 4 °C, the upper layer was removed to obtain the LPS pellet. The pellet was washed with 70% ethanol and centrifuged at 13,000 rpm for 3 min at 4 °C. After discarding the resulting upper layer, the pellet was dried at room temperature and dissolved in 10 mM Tris-HCl (pH 8.0) by boiling for 2 min. To get the pure LPS from the VPGs, proteinase K (2.5 μg/LPS 1 μg) was added to the dissolved LPS and the mixture was incubated at 4 °C for 30 min. Finally, the purified LPS (5 μL) was loaded for SDS-PAGE analysis. LPS extracted from the untreated bacterial cells was included as a positive control. The gel was stained in silver solution according to Fomsgaard et al. [51]. In addition, standard LAL assay has been used to evaluate the endotoxic activity by using Pierce LAL Chromogenic Endotoxin Quantitation Kit (Thermo Fisher Scientific, Waltham, MA, USA), according to the manufacturer’s instructions. *E*. *coli* 011:B4 LPS was used as a standard and results were given in EU.

### 3.6. Assessment of Macrophage-Mediated Cytotoxicity

Murine macrophage (KCLB:40071, RAW 264.7) cells were purchased from Korean Cell Line Bank (Seoul, Korea) and cultured in 96-well plates (BD Falcon; BD Bioscience Discovery Labware, Bedford, MA, USA) for 24 h at 37 °C, in humidified 5% CO_2_, 95% air. The cells (1.5 × 10^3^ cells/well) were then treated with various doses (2.5, 1.7, 1.3, 1.0, and 0.5 × 10^6^ CFU/mL) of the NaOH-induced VPGs and wild-type cells in culture medium, and incubated for a further 24 h. PBS treated- and LPS (5 μg/mL)-treated macrophages were used as controls. The cell density was then assessed by using Cell Counting Kit-8 (CCK-8, Sigma-Aldrich, St. Louis, MO, USA) analysis. Absorbance was measured at 450 nm and all experiments were performed in triplicate. Cytotoxic activity is expressed as the percentage of cell viability by the following formula: %Cytotoxicity = (1 − A_450nm_ of target cells/A_450nm_ of control cells) × 100.

### 3.7. Quantitative Analysis of Cytokine mRNA by Reverse Transcription (RT)-qPCR

RAW 264.7 cells (1.5 × 10^3^ cells/well) were cultured in 24-well flat-bottom plates and treated with the wild-type cells and VPGs, respectively, at a concentration of 0.5 × 10^6^ CFU/mL. After 24 h stimulation, total RNA was isolated using RNAiso (Takara Bio, Shiga, Japan), according to the manufacturer’s instructions. TNF-α, IL-1β, IL-6, IL-10, IL-12, and iNOS mRNA levels were quantified by RT-qPCR amplification. Sequences for the primers of target genes are listed in Table 2. RT reaction was performed in a 20 μL reaction mixture containing 300 ng of total RNA, 50 mM Tris-HCl (pH 8.3), 75 mM KCl, 8 mM MgCl_2_, 10 mM DTT, 0.1% NP-40, 40 mM dNTP, 2 pM of respective primer set, 20 U of RNase inhibitor (Takara Bio), and 200 U PrimeScript Reverse Transcriptase (Takara Bio). The thermal cycler was programmed for 1 RT cycle at 50 °C for 30 min and 70 °C for 15 min. cDNA was amplified in a 20 μL reaction mixture containing 10 μL 2X SYBR^®^ Premix Ex Taq™ II (Tli RNaseH Plus, Takara Bio), 0.2 μL ROX reference dye II, 0.4 μL of 10 μM of both forward and reverse primer (Table 2), and 1 ng of cDNA, using Stratagene Mx3005P cycler (1 cycle at 95 °C for 30 s, 30 cycles of denaturation at 95 °C for 5 s, and primer annealing and extension at 60 °C for 34 s). Each gene was amplified in triplicate and cDNA concentration differences were normalized to glyceraldehyde 3-phosphate dehydrogenase (GAPDH).

### 3.8. SEM, SDS-PAGE, and Agarose Gel Electrophoresis Analyses

Morphological analysis of the NaOH-induced VPGs was performed by SEM, as previously described [16,17]. Both the NaOH-induced VPGs and untreated control bacterial cells were denatured in Laemmli’s buffer [52] and then loaded in 12% SDS-PAGE under constant 40 mA. In addition, the culture supernatants were precipitated using ammonium sulfate (final conc. 60%) for the extracellular protein concentration. The gel was stained in Coomassie brilliant blue solution for 4 h at room temperature and immersed in destaining solution (45% methanol; 10% acetic acid; 45% distilled water). The protein concentration was determined by using a Bio-Rad protein assay. To confirm the absence of genomic DNA in the NaOH-induced VPGs, genomic DNA was isolated by using a bacterial genomic DNA isolation kit (iNtRON Biotechnology). To determine the presence of DNA released extracellularly, DNA was concentrated from the culture supernatants using 2 vol (*v*/*v*) of ethanol and 1/10 vol (*v*/*v*) of sodium acetate and analyzed using 1% agarose gel.

### 3.9. Statistical Analysis

Data were analyzed for statistical significance by the SPSS software (version 21.0), and means were compared using Duncan’s multiple range tests. Graphing was conducted with SigmaPlot 12.5 (Systat Software, Inc., San Jose, CA, USA).

## 4. Conclusions

NaOH has proven as the best chemical to affect *V*. *parahaemolyticus* cell walls and its MIC successfully created trans-membrane lysis channels on the surface of VPGs. Eventually, *Vibrio* cells were devoid of cytoplasmic and genetic contents, and turned into empty cell envelopes. LPS extracted from the wild-type bacterial cells showed triple major bands on the SDS-PAGE gel after silver staining, while LPS extracted from the NaOH-induced VPGs showed no distinctive band. It suggests that some surface structures in the LPS may be lost, weakened, or modified by the treatment of NaOH at MIC. Nevertheless, the LAL test showed that there was no significant difference in endotoxic activity between the VPGs and wild-type bacterial cells. Macrophages exposed to the VPGs at 0.5 × 10^6^ CFU/mL showed cell viability of 97.9%, though the MIC of NaOH did not reduce the cytotoxic effect. Like the *E. coli* LPS, the VPGs are an excellent activator of pro-inflammatory cytokines such as IL-1β and iNOS, anti-inflammatory cytokine IL-10, and dual activities of IL-6 in the stimulated macrophages, when compared with wild-type bacterial cells. On the other hand, wild-type bacterial cells showed the highest induction of TNF-α mRNA in the exposed macrophages. All these results indicate that NaOH-induced VPGs show the potency of a safe, economical, and effective inactivated vaccine candidate.

## Figures and Tables

**Table 1:** Minimum inhibitory concentration (MIC) of chemicals treated into *V*. *parahaemolyticus* culture medium.

Chemical	MIC (mg/mL)	Medium pH
Sodium hydroxide (NaOH)	3.125	9.99
Acetic acid (CH_3_COOH)	6.25	4.28
Boric acid (BH_3_O_3_)	<50	-
Citric acid (C_6_H_8_O_7_)	25	3.11
Hydrochloric acid (HCl)	1.56	3.95
Maleic acid (C_4_H_4_O_4_)	6.25	3.56
Sulfuric acid (H_2_SO_4_)	0.781	5.83

**Table 2:** Primer sequences and fragment sizes of the targeted genes in real-time PCR.

Gene	Orientation	Primer Sequences (5′–3′)
16s rRNA	forward	ATATGCCTAGGTGGGATTAGCTAGT
	reverse	TGTCTCAGTTCCAGTGTGGCTG
TNF-α	forward	ATGAGCACAGAA AGCATGATCCG
	reverse	GCTGAGACATAGGCACCGC
Il-1β	forward	ATGGCAACTGTTCCTGAACTCAACT
	reverse	AGTAGCCCTTCATCTTTTGGGG
IL-6	forward	ATGAAGTTCCTCTCTGCAAGAGACT
	reverse	GTCTCCTCTCCGGACTTGTGA
IL-10	forward	ATGCCTGGCTCAGCACTGCTA
	reverse	CTGGGAAGTGGGTGCAGTTATTG
IL-12	forward	ATGTGTCAATCACGCTACCTCCT
	reverse	GACTGGCTAAGACACCTGGC
iNOS	forward	ATGAACCCCAAGAGTTTGACCAGA
	reverse	GGAGCCATAATACTGGTTGATGAAC
GAPDH	forward	ATGGTGAAGGTCGGTGTGAACG
	reverse	CAATGAAGGGGTCGTTGATGGC

## Supplementary Materials

Supplementary materials can be found at www.mdpi.com/1422-0067/17/11/1904/s1.
