# Supplementary material for: Characterization of Chemically-Induced Bacterial Ghosts (BGs) Using Sodium Hydroxide-Induced Vibrio parahaemolyticus Ghosts (VPGs)
Source: Int J Mol Sci. 2016 Nov 15;17(11):1904. doi: 10.3390/ijms17111904 (PMC5133902; doi:10.3390/ijms17111904)
Supplement: Supplementary file 1 [file ijms-17-01904-s001.pdf]

Supplementary Material: Characterization of Chemically-Induced Bacterial ghosts (BGs) Using Sodium Hydroxide-Induced *Vibrio parahaemolyticus* Ghosts (VPGs)

Hyun Jung Park, Sung Oh, Nagarajan Vinod, Seongmi Ji, Han Byul Noh, Jung Mo Koo, Su Hyeong Lee, Sei Chang Kim, Ki-Sung Lee and Chang Won Choi

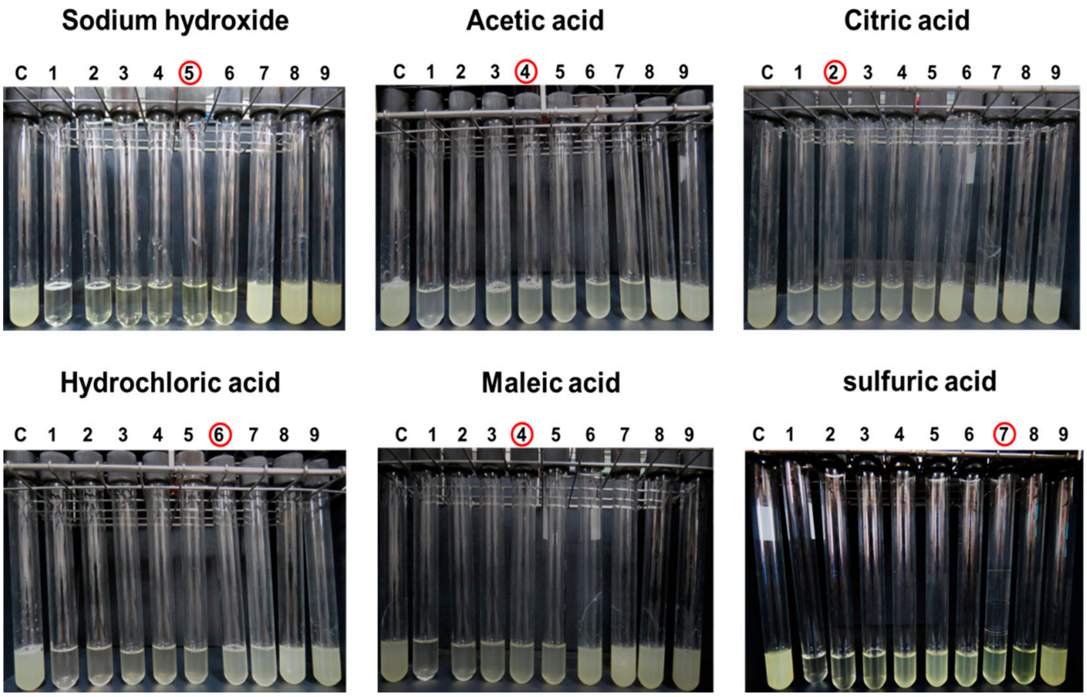

Figure S1. Determination of MIC of *V. parahaemolyticus* PCU-1 bacterial cells treated with various chemicals, respectively. Red circle indicates the MIC of each chemical. Tubes 1–9 represent bacterial culture tube treated with respective chemicals at 50, 25, 12.5, 6.25, 3.125, 1.563, 0.781, 0.391 and 0.195 mg/mL and tube C (none-treated control).

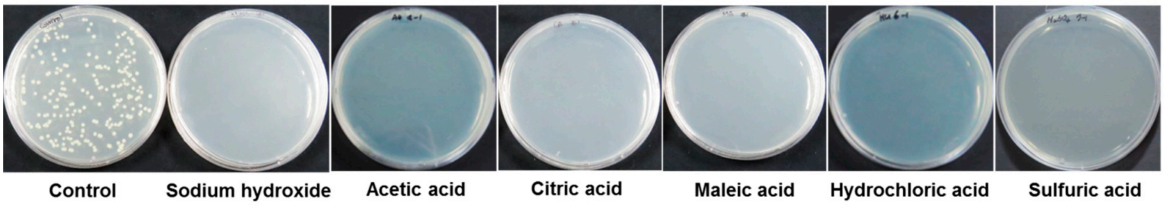

Figure S2. MIC of various chemicals against *V. parahaemolyticus* PCU-1 bacterial cells showed no viability.

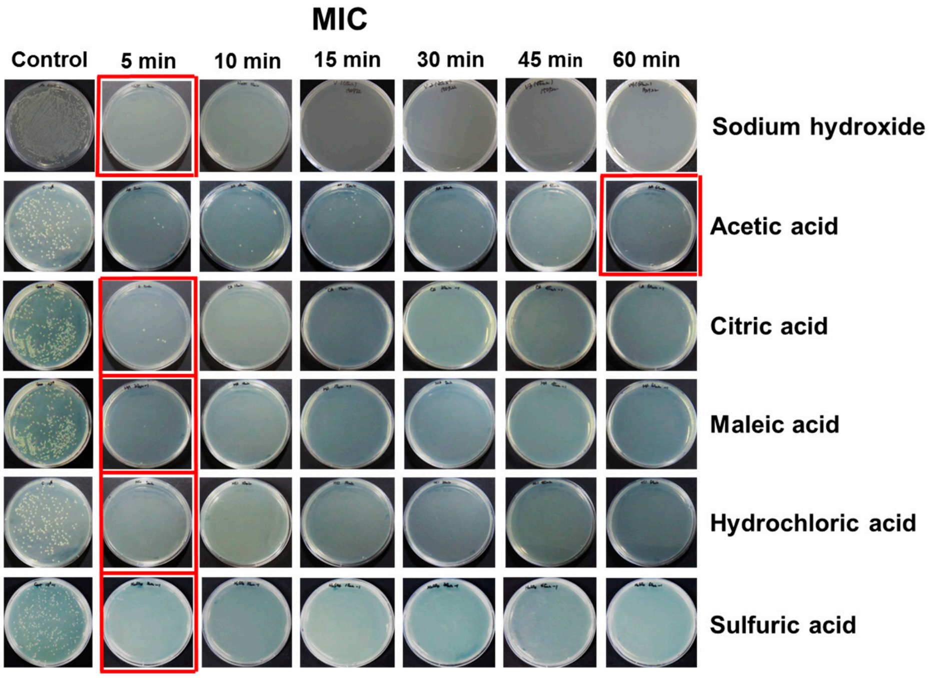

Figure S3. Determination of VPGs viability. From left, non-treated control *V. parahaemolyticus* PCU-1 bacterial cells (C) and NaOH MIC-induced VPGs after 15, 30, 45 and 60 min.
